# NEIGHBORHOOD ENVIRONMENT AND PHYSICAL ACTIVITY AMONG PERSONS WITH DEMENTIA

**DOI:** 10.1093/geroni/igae098.1379

**Published:** 2024-12-31

**Authors:** Eunyoung Choi, Yeon Jin Choi, Hyungmin Cha, Calley Fisk, Rachel Wilkie, Jennifer Ailshire

**Affiliations:** University of Southern California, Los Angeles, California, United States; University of Kentucky, Lexington, Kentucky, United States; University of Southern California, Los Angeles, California, United States; University of Southern California, Los Angeles, California, United States; University of Southern California, Los Angeles, California, United States; University of Southern California, Los Angeles, California, United States

## Abstract

Physical activity plays an important role in delaying the progression of cognitive and functional decline among people with dementia (PWD). Neighborhood environments can provide key resources or function as critical barriers to promoting physical activity among PWD. However, the specific neighborhood factors that drive this relationship are yet to be determined. Our study examines how socioeconomic (i.e., area affluence and disadvantage), physical (i.e., annual average PM2.5, temperature, park area), and social (i.e., cohesion, disorder, and safety) aspects of neighborhood environment are associated with physical activity levels among PWD living in communities across the U.S. We used data from 2006 to 2018 of Health and Retirement Study for dementia status and neighborhood social environment, merged with census-tract level data from the Contextual Data Resource for socioeconomic and physical neighborhood factors. Specifically, we used the first wave of data where PWD were identified as having dementia (N=3,073). Physical activity was measured as a weighted sum of light, moderate, and vigorous activity (range: 0-31). We estimated linear regression models adjusting for individual-level sociodemographic covariates. Higher neighborhood socioeconomic affluence (B=0.45, 95% CI=0.07, 0.84) and social cohesion (B=0.70, 95% CI=0.32, 1.09) were associated with higher levels of physical activity. Conversely, any sign of neighborhood physical disorder (B=-1.23, 95%CI=-2.09, -0.38) was associated with reduced physical activity. No significant relationships were found from physical neighborhood environment factors. This study provides empirical evidence for the social-ecological model which posits the important role of broader environmental factors that influence PWD’s physical activity participation.

